# Blunt popliteal artery injury following tibiofemoral trauma: vessel-first and bone-first strategy

**DOI:** 10.1007/s00068-021-01632-0

**Published:** 2021-03-20

**Authors:** Dennis Hundersmarck, Falco Hietbrink, Luke P. H. Leenen, Gert J. De Borst, Marilyn Heng

**Affiliations:** 1 Department of Orthopedic Surgery, Harvard Medical School Orthopedic Trauma Initiative, Massachusetts General Hospital, 55 Fruit Street, Boston, USA; 2 Department of Surgery, University Medical Center Utrecht, Utrecht, The Netherlands; 3 Department of Vascular Surgery, University Medical Center Utrecht, Utrecht, The Netherlands

**Keywords:** Blunt popliteal artery injury, Popliteal artery injury, Knee dislocation, Tibiofemoral trauma, Vascular injury

## Abstract

**Purpose:**

Blunt popliteal artery injury (BPAI) is a potentially limb-threatening sequela of tibiofemoral (knee) dislocations and fractures. Associated amputation rates for all popliteal artery (PA) injuries range between 10 and 50%. It is unclear whether PA repair or bone stabilization should be performed first. We analyzed (long-term) clinical outcomes of BPAI patients that received initial PA repair (vessel-first, VF) versus initial external stabilization (bone-first, BF).

**Methods:**

Retrospectively, all surgically treated BPAI patients between January 2000 and January 2019, admitted to two level 1 trauma centers were included. Clinical outcomes were determined, stratified by initial management strategy (VF and BF). Treatment strategy was determined by surgeon preference, based on associated injuries and ischemia duration. Primary outcomes (amputation and mortality) and secondary outcomes (claudication and complications) were determined.

**Results:**

Of 27 included BPAI patients, 15 were treated according to the VF strategy (56%) and 12 according to the BF strategy (44%). Occlusion was the most frequently encountered BPAI in 18/27 patients (67%). Total delay and in-hospital delay were comparable between groups (*p* = 1.00 and *p* = 0.82). Revascularization was most frequently performed by PA bypass (59%). All patients had primary limb salvage during admission (100%). One secondary amputation due to knee pain was performed in the BF group (4%). During a median clinical follow-up period of 2.7 years, three PA re-interventions were performed, two in the BF group and one in the VF group. None suffered from (intermittent) claudication.

**Conclusion:**

Blunt popliteal artery injury (BPAI) is a rare surgical emergency. Long-term outcomes of early revascularization for BPAI appear to be good, independent of initial management strategy. The BF strategy may be preferred in case of severe orthopedic injury, if allowed by total ischemia duration.

**Supplementary Information:**

The online version contains supplementary material available at 10.1007/s00068-021-01632-0.

## Introduction

Blunt popliteal artery injury (BPAI) is a potentially limb-threatening sequela of tibiofemoral (knee) dislocations and fractures. The reported incidence of concomitant BPAI in patients with knee dislocations varies widely, ranging from 2% among those that require surgical repair of the popliteal artery in a population-based study to 40% of consecutive knee dislocations at a single tertiary referral institution [1, 2]. Reported associated amputation rates are equally varying, ranging between 10 and 50% [3–5]. Low-reported incidence rates, combined with the large potential for limb loss, make BPAI a rare and challenging surgical emergency.

As BPAIs disrupt distal blood flow above the trifurcation, causing ischemia of the lower leg due to the absence of a significant collateral circulation at this level, early identification and treatment of knee dislocation patients with concomitant arterial injury is important. Prompt reduction of knee dislocations and careful vascular examination, using computed tomography angiography (CTA) scanning in those suspected for vascular injury, is required [6–8]. Specifically, those that show signs of vascular impairment prior to reduction and apparent return of vascular patency after reduction are at risk for latent ischemia (due to dissections) and amputation [9]. It is reported that a warm-ischemia period of 8 h or more will most likely result in amputation [10].

In those that require surgical treatment of BPAIs, it is unclear whether repair of the arterial disruption or bone stabilization should be performed first [11, 12]. The so-called vessel-first (VF) strategy advocates lower leg revascularization by definite popliteal artery repair, preceded by intraluminal shunting if applicable (e.g., during vein graft harvesting), before temporary bone stabilization is performed, followed by definite orthopedic stabilization in a later stage [13]. Potential benefits of this treatment strategy are minimizing ischemia times and optimizing surgical exposure due to the absence of external fixation materials.

However, as knee dislocation patients with BPAIs usually have severe or total ligamentous disruption of the knee that causes significant tibiofemoral translation, initial temporary external fixation may be preferred to protect a vascular repair from traction injuries [14, 15]. In addition, this so-called bone-first (BF) strategy may be beneficial in providing reduction of (severely bleeding or sharp) fracture elements of associated tibial plateau and distal femur fractures, in order to protect a vascular graft. Furthermore, as a BF strategy improves osseous alignment, this may lead to improved positioning and adequate lengthening of a vascular graft. As data on these management strategies for BPAI are limited, the results of a BF strategy and the comparison between both strategies remains unknown.

This study compares patients that received BPAI treatment according to a VF strategy to those that received treatment according to a BF strategy, and aims to determine if a BF strategy will lead to increased (ischemic) complication rates.

## Methods

### Patient selection

All surgically treated BPAI patients ≥ 18 years old with concomitant tibiofemoral dislocations and fractures, admitted from the emergency department at two level 1 trauma centers between January 2000 until January 2019, were included in this study. The institutional research patient data registry (RPDR) was queried for corresponding International Classification of Disease (ICD) 9 and 10 codes for popliteal artery injury and tibiofemoral dislocations and fractures. Patients younger than 18 years old, those with incomplete medical records, those with penetrating popliteal artery injury, and those that did not undergo revascularization (e.g., because of mangled extremities that required primary amputation) were excluded.

### Patient characteristics

Demographic and clinical patient characteristics were extracted from medical records (Table 1). Knee dislocations were graded based on the Schenck classification, and tibial plateau fractures were graded based on the Schatzker classification, both using available radiological images and reports and clinical data [16, 17]. BPAI grade was determined based on computed tomography angiography (CTA) imaging and radiology reports. Lower leg ischemia was determined according to the Rutherford grading scale for acute limb ischemia, using clinical notes [18]. Due to the limitations of retrospectively reviewing clinical notes for determining the Rutherford sub classification [marginally threatened (IIa) and immediately threatened (IIb)], we limited classification of acute extremity ischemia to three grades: not immediately threatened (I), threatened (II, combining the sub classification), and irreversible (III). Total delay duration was defined as time between the reported time of injury and operating room arrival time. In-hospital delay duration was defined as time between the initial encounter noted in the emergency department records and operating room arrival time, including patients that received revascularization at a different hospital. Infectious complications were defined as surgical site infections or osteosynthesis-related infections. Thrombo-embolic complications were defined as new pulmonary embolisms or deep venous thrombo-embolisms detected on imaging while admitted to hospital. The decision to either perform an initial vascular repair or initial external fixation was made according to preference of the attending surgeon, based on the total injury burden, extent of associated orthopedic injuries, and the total delay duration. In case of isolated extremity injury in the absence of severe associated injuries and in case of polytrauma patients requiring a damage-control approach, a VF strategy was preferred. If severe associated orthopedic injury or knee instability was present, a BF strategy was preferred if allowed by the total ischemia duration. To illustrate potential differences between patients that were transferred from scene versus those that were transported from other hospitals, a comparison between these patients is displayed in the supplementary materials Tables 1, 2 and 3.

**Table 1:** Characteristics of all patients, those treated by vessel-first strategy, and those treated by bone-first strategy

	All patients (*n* = 27)	Vessel-first (*n* = 15)	Bone-first (*n* = 12)	*p* value
Age (years)	38 [22–54]	34 [20–51]	47 [29–58]	0.25
Male	15 (56)	7 (47)	8 (67)	0.30
BMI (kg/m^2^)	36 [28–48]	37 [32–70]	30 [25–44]	0.11
Systolic blood pressure	133 [118–155]	134 [118–155]	130 [114–156]	0.62
Hemodynamic instability	4 (15)	1 (7)	3 (25)	0.29
Absent pulsations	25 (93)	13 (87)	12 (100)	0.19
Inaudible doppler signal	19 (70)	9 (60)	10 (83)	0.19
ISS	9 [4–10]	4 [4–14]	9 [4–10]	0.37
Polytrauma	5 (19)	3 (20)	2 (17)	1.00
Hb (mmol/L)	8.3 [7.4–8.7]	8.3 [7.4–9.1]	8.0 [7.4–8.6]	0.85
OSH ED presentation	16 (59)	10 (67)	6 (50)	0.38
OSH vascular repair	2 (7)	1 (7)	1 (8)	1.00
Total delay (hours)*	5.7 [4.0–8.0]	5.5 [4.0–8.5]	6.8 [4.0–7.5]	1.00
In-hospital diagnostic delay (hours)	2.7 [2.0–3.0]	2.6 [2.0–2.9]	2.7 [1.0–3.0]	0.82
Severity of limb ischemia**				0.64
Viable (I)	6 (22)	4 (27)	2 (17)	
Threatened (II)	18 (67)	10 (67)	8 (67)	
Irreversible (III)	3 (11)	1 (7)	2 (17)	

Data are presented as the number (%) or the median [IQR: 25th–75th percentile]

*BMI* body mass index, *ISS* Injury Severity Score, *Hb* hemoglobin, *OSH ED* outside-hospital emergency department

*Total delay duration could not be accurately determined in 6 vessel-first patients and 2 bone-first patients

**Using the (modified) Rutherford classification for acute limb ischemia. Percentages may not be total 100 due to rounding

### Follow-up

All out-patient encounters of all identified patients were reviewed, in order to determine postdischarge outcomes. Time to follow-up was defined as time between hospital discharge and most recent out-patient visit, rather than a standardized follow-up moment. Vascular re-interventions were defined as all surgical or endovascular procedures performed to restore vascular patency. Claudication was defined as (intermittent) claudication complaints of the affected leg, excluding those with vascular re-interventions.

### Statistical analysis

Data were analyzed using STATA^®^ statistical software (StataCorp. 2017. Release 15. College Station, TX: StataCorp LLC.) Baseline and clinical patient characteristics were, in the case of non-parametrical distributed categorical explanatory variables and dichotomous outcome variables, analyzed using the two-sided Chi-square test or two-sided Fisher’s exact test. In the case of dichotomous explanatory variables and continuous outcome variables, the Mann–Whitney *U* test was used. In the case of multiple non-parametric categorical explanatory variables and continuous outcome variables, the Spearman-Rank correlation was used. A *p* value of < 0.05 was considered significant.

## Results

### Patient and injury demographics

Twenty-seven patients with BPAI that met the inclusion criteria were identified and included. One patient with a penetrating popliteal artery injury was excluded. Three patients that had BPAI and did not undergo arterial repair were excluded. Two of these patients had mangled extremities that were deemed unsalvageable, requiring primary amputation. One patient was transferred from a different hospital approximately 17 days after the initial knee injury, latent ischemia caused by a previously undiagnosed popliteal artery dissection resulted in primary amputation. Baseline characteristics of all 27 included patients, the 15 patients that received primary arterial repair first (VF, 56%), and the 12 patients that received primary external fixation first (BF, 44%) are depicted in Table 1. Median (IQR) body mass index (BMI) was 36 (28–48) and the most common mechanism of injury was a low-energy fall from height less than 2 m in eight cases (30%) followed by crush injuries in seven cases (26%). As 5/27 patients were polytrauma patients (19%) and median (IQR) ISS was 9 (4–10), most patients suffered from isolated lower extremity injuries. Sixteen patients initially presented at a different hospital (59%), of which two received vascular repair at that hospital prior to transfer to our institution (7%). All patients showed signs of lower leg ischemia, of which most legs were threatened (67%). Twenty-three patients had knee dislocations (85%), of which seven (26%) had Schenck grade V injuries (multiligamentous injury with peri-articular fracture). Nine patients had associated tibial plateau fractures of which most had Schatzker type 4 fractures (*n* = 4), followed by Schatzker type 6 fractures (*n* = 3). The most common popliteal artery injuries were occlusions in 67% of cases. Injury mechanisms and associated injuries are depicted in Table 2.

**Table 2:** Injury characteristics of all patients, those treated by vessel-first strategy, and those treated by bone-first strategy

	All patients (*n* = 27)	Vessel-first (*n* = 15)	Bone-first (*n* = 12)	*p* value
Mechanism of injury				0.29
Fall from height (< 2 m)	8 (30)	6 (40)	2 (17)	
Crush/impact	7 (26)	2 (13)	5 (42)	
Fall from height (> 2 m)	3 (11)	3 (20)	0 (0)	
Pedestrian collision	3 (11)	1 (7)	2 (17)	
MVA	2 (7)	1 (7)	1 (8)	
Other	4 (15)	2 (13)	2 (17)	
Schenck knee dislocation grade*				0.67
I	0 (0)	0 (0)	0 (0)	
II	3 (11)	1 (7)	2 (17)	
III	6 (22)	4 (27)	2 (17)	
IV	2 (7)	1 (7)	1 (8)	
V	7 (26)	5 (33)	2 (17)	
N/a	4 (15)	1 (7)	3 (25)	
Popliteal artery injury				0.26
Dissection	3 (11)	3 (20)	0 (0)	
Occlusion	18 (67)	9 (60)	9 (75)	
Transection	6 (22)	3 (20)	3 (25)	
Previous knee injury	5 (19)	2 (13)	3 (25)	0.44
Knee dislocation**	23 (85)	14 (93)	9 (75)	0.18
Isolated ligamentous injuries	14 (52)	8 (53)	6 (50)	0.86
Tibial plateau fracture	9 (33)	5 (33)	4 (33)	1.00
Schatzker classification				0.35
Type 3	1 (1)	1 (7)	0 (0)	
Type 4	4 (15)	3 (20)	1 (8)	
Type 5	1 (4)	0 (0)	1 (8)	
Type 6	3 (11)	1 (7)	2 (17)	
Additional knee injuries				
Distal femur fracture	4 (15)	2 (13)	2 (17)	1.00
Fibula fracture	6 (22)	4 (27)	2 (17)	0.66
Popliteal vein injury	2 (7)	0 (0)	2 (17)	0.19
Tibial nerve injury	1 (4)	1 (7)	0 (0)	1.00

Data are presented as the number (%) or the median [IQR: 25th–75th percentile]

*MVA* motor vehicle accident. Percentages may not total 100 due to rounding

*Due to the absence of MRI imaging records, Schenck knee dislocation grade could not be determined in five patients

**Including fracture dislocations

### Vessel- and bone-first strategy-related outcomes

Median (IQR) total delay duration in the VF group was 5.5 (4.0–8.5) [95% confidence interval (CI) 4.0–9.9] h and median (IQR) total delay duration in the BF group was 6.8 (4.0–7.5) (95% CI 3.8–7.8) h. Median (IQR) in-hospital delay duration in the VF group was 2.6 (2.0–2.9) (95% CI 2.0–3.0) h and median (IQR) in-hospital delay duration in the BF group was 2.7 (1.0–3.0) (95% CI 0.8–3.2) h. Both total delay duration and in-hospital delay duration were not significantly different between groups (*p* = 1.00 and *p* = 0.85), as is depicted in Table 1. Median (IQR) total delay duration was higher in those that initially presented at different hospitals compared to those that initially presented at the studied institutions [7.5 (5.7–10.0) vs. 4.0 (3.8–4.6) hours; *p* < 0.01] which is depicted in supplementary materials, Table 1.

Eight patients in the VF group had isolated ligamentous injuries (53%), compared to six in the BF group (50%), which was not different between groups (*p* = 0.86). Five patients in the VF group had tibial plateau fractures (33%), compared to four in the BF group (33%), which did not differ between groups (*p* = 1.00), as is depicted in Table 2. The most commonly observed Schatzker type of tibial plateau fracture in the VF group was type 3 (*n* = 3), and in the BF group, this was type 6 (*n* = 2) (*p* = 0.35). No primary amputations were observed. The only observed secondary amputation was observed in the BF group. This patient initially had successful revascularization approximately 7.5 h after the initial injury and was discharged after 5 days. During follow-up, the patient suffered from an iatrogenic injury to the popliteal artery bypass during meniscal surgery and ligament reconstruction at a different hospital. This resulted in multiple vascular re-interventions to restore patency, after which eventually an above the knee amputation was performed due to chronic pain and knee instability (approximately 5.5 years after initial presentation). Because this secondary amputation was the result of an iatrogenic injury rather than the initial management strategy, this patients was excluded for this analysis. No in-hospital mortalities were found. Two patients in the VF group had local infections, compared to one in the BF group (*p* = 0.54). Twelve patients in the VF group received fasciotomies (80%), compared to 10 in the BF group (83%, *p* = 0.83). Nineteen out of 22 fasciotomies were prophylactic (86%). No thrombo-embolic complications were observed. No differences in terms of studied outcomes were observed when comparing those that presented at other hospitals to those that initially presented at the studied institutions (supplementary materials, Table 3).

### Vascular repair details

Vascular repair details and outcomes of both groups are depicted in Table 3. In 16 patients, revascularization was achieved by popliteal artery bypass (59%), followed by interposition grafts in nine patients (33%) and endovascular repair in two patients (7%). All popliteal artery bypasses were performed using reversed greater saphenous vein (GSV) grafting. Two interposition grafts were performed using reversed GSV and the rest utilized synthetic graft materials. Intraluminal shunting was used during surgery in two patients (7%), of which one shunt occluded. This patient was treated by initial external stabilization (BF) and intraluminal shunting. Shortly after the initial surgery, shunt occlusion occurred, requiring immediate popliteal artery bypass.

**Table 3:** Treatment characteristics and outcomes of all patients, those treated by vessel-first strategy, and those treated by bone-first strategy

	All patients (*n* = 27)	Vessel-first (*n* = 15)	Bone-first (*n* = 12)	*p* value
Popliteal artery reconstruction				1.00
Bypass	16 (59)	9 (60)	7 (58)	
Interposition graft	9 (33)	5 (33)	4 (33)	
Endovascular stenting	2 (7)	1 (7)	1 (8)	
Used reconstruction materials				0.42
Greater saphenous vein	23 (85)	12 (80)	11 (92)	
Synthetic graft	2 (7)	2 (13)	0 (0)	
Stent graft	2 (7)	1 (7)	1 (8)	
Surgical approach				
Preoperative angiography	14 (52)	9 (60)	5 (42)	0.45
Medial approach	23 (85)	13 (87)	10 (83)	0.49
Posterior approach	3 (11)	2 (13)	1 (8)	0.77
Endovascular	2 (7)	1 (7)	1 (8)	1.00
Proximal anastomosis site				0.68
PA	16 (59)	10 (67)	6 (50)	
SFA	9 (33)	5 (33)	4 (33)	
N/a	2 (7)	1 (7)	1 (8)	
Distal anastomosis site				0.72
PA	19 (70)	11 (73)	8 (67)	
PTA	5 (19)	2 (13)	3 (25)	
TPT	1 (4)	1 (7)	0 (0)	
N/a	2 (7)	1 (7)	1 (8)	
Additional procedures				
Fasciotomy	22 (81)	12 (80)	10 (83)	0.83
Shunting	2 (7)	1 (7)	1 (8)	1.00
Additional orthopedic treatment				
ORIF	6 (22)	2 (13)	4 (33)	0.21
Ligament reconstruction	8 (30)	5 (33)	3 (25)	0.67
Postoperative medical treatment				0.48
ASA	20 (74)	12 (80)	8 (67)	
Coumadin	1 (4)	0 (0)	1 (8)	
None	6 (22)	3 (20)	3 (25)	
Outcomes				
Primary amputations	0 (0)	0 (0)	0 (0)	1.00
Secondary amputations	0 (0)	0 (0)	0 (0)	1.00
Preoperative compartment syndrome	3 (11)	2 (13)	1 (8)	1.00
Postoperative compartment syndrome	0 (0)	0 (0)	0 (0)	1.00
Thrombo-embolic complications	0 (0)	0 (0)	0 (0)	1.00
Postoperative infections	3 (11)	2 (13)	1 (8)	0.57
Mortality	0 (0)	0 (0)	0 (0)	1.00
Median (IQR) LOS (days)	12 [7–21]	12 [6–14]	16 [10–28]	0.12
Median (IQR) ICU-LOS (days)	0.5 [0–3]	0 [0–1]	1 [0–4]	0.21
Follow-up				
Available follow-up	25 (93)	13 (87)	12 (100)	0.49
Median (IQR) time to follow-up (years)	2.7 [1.1–5.9]	1.4 [0.6–3.0]	3.5 [1.6–7.5]	0.14
Popliteal artery re-intervention	3 (11)	1 (7)	2 (17)	0.57
Claudication	0 (0)	0 (0)	0 (0)	1.00

Data are presented as the number (%) or the median [IQR: 25th–75th percentile]

*PA* popliteal artery, *SFA* superficial femoral artery, *PTA* posterior tibial artery, *TPT* tibioperoneal trunk, *ORIF* open reduction internal fixation, *ASA* acetylsalicylic acid, *LOS* length of stay, *ICU* intensive-care unit

Percentages may not total 100 due to rounding

The popliteal artery was the most common proximal anastomosis site (59%) as well as the most common distal anastomosis site (70%). The two observed popliteal vein injuries, a traumatic occlusion and a transection, were treated conservatively and by ligation, respectively. Neither injury resulted in complication.

### Follow-up and long-term outcomes

Twenty-five patients were available for follow-up during out-patient visits (93%). During the median (IQR) follow-up period of 2.7 years (1.1–5.9), three patients required ipsilateral popliteal artery re-interventions to restore vascular patency. One of these patients experienced acute occlusion of the polytetrafluoroethylene (PTFE) interposition graft, while on acetylsalicylic acid (ASA) 325 mg, vascular patency was restored by percutaneous transluminal angioplasty and stent placement. The second patient experienced a proximal stenosis of the GSV bypass, requiring vein patch angioplasty after which vascular patency was restored. The third patient is the previously described patient that experienced an iatrogenic injury to the popliteal artery bypass during a subsequent surgery and then required multiple vascular re-interventions ultimately resulting in amputation due to pain. In total, 20 patients were started on ASA medication (in most cases dosed 325 mg once daily) to prevent thrombus formation and occlusion of the vascular graft (74%). After exclusion of the patients that required vascular re-interventions, no patients experienced claudication complaints at long-term follow-up.

## Discussion

In this retrospective study, we determined (long-term) clinical outcomes of patients that underwent BPAI repair following tibiofemoral trauma. Taking the number of observed BPAI patients, the studied 19-year period in two level 1 trauma centers and the large potential for limb loss into account, we determined that BPAI remains a rare and challenging surgical emergency. We compared outcomes of patients that received initial vascular repair (VF) to those that received initial temporary external stabilization of fractures and dislocations (BF) and found an overall primary limb salvage rate of 100% with comparable outcomes between the VF and BF groups.

### Vessel-first and bone-first strategy

International guidelines regarding the management of lower extremity trauma do not provide unambiguous recommendations whether revascularization or bone stabilization should be performed first in tibiofemoral trauma patients with popliteal artery injury [12, 19]. The Western Trauma Association guideline recommendation regarding management of the mangled extremity states that in hemodynamically stable patients, initial reduction of fractured bones using either splinting or traction fixation will alleviate kinking of the vasculature and improve subsequent perfusion. However, in case of vascular injury in addition to orthopedic injury in patients with isolated extremity injuries or limited associated injuries, no recommendations regarding a VF or BF strategy are made. In case of hemodynamic instability and an extremity which is deemed salvageable, initial use of an intraluminal shunt is recommended [12].

As multiple studies emphasize the influence of total ischemia duration on clinical outcomes, initial revascularization is often preferred [10, 20]. This VF strategy has been associated with good clinical outcomes [21]. The absence of external fixation materials during a VF strategy may provide better popliteal artery exposure during surgery. During a damage-control approach in polytrauma patients, a VF strategy (utilizing intraluminal shunting) may be preferable as it reduces operating time. However, we speculate that initial bone stabilization (BF) may be preferred in patients with severe isolated orthopedic injuries, to prevent traction injuries to the vascular graft and to prevent sharp fracture elements from damaging the vascular graft. Furthermore, tibiofemoral alignment may aid the correct positioning and lengthening of the vascular graft.

In addition to current literature, we were able to compare both the VF and BF strategy in BPAI patients (mostly consisting of isolated lower extremity trauma patients) and determined that a BF strategy could be safely performed in those where primary osseous stabilization was deemed necessary.

Although a few studies have described external fixation before or after popliteal artery repair, the studied patients suffered mostly from penetrating (popliteal) arterial injuries in addition to blunt injuries [22, 23]. Neither specifically studied popliteal artery injuries, since all lower extremity vascular injuries were combined. One of these studies, consisting of 17 lower extremity vascular injuries, made a recommendation regarding the optimal strategy, advocating initial revascularization over orthopedic stabilization [22]. A retrospective study focusing strictly on blunt injuries to the popliteal artery identified multiple factors associated with limb salvage, of which prehospital delay > 6 h was frequent among amputees, but did not study the effects of a VF or BF strategy [24].

Penetrating and blunt injuries to the popliteal artery result in different types of arterial damage (e.g., transection with free extravasation and tourniquet application vs. dissections and near-occlusions), caused by different injury mechanisms (e.g., knife/gunshot wounds vs. mechanical falls/crush injuries) with different associated injuries. Of these, BPAIs are associated with a higher frequency of secondary amputations, due to higher-energy trauma [20]. Due to these differences in both the arterial injury and the associated injuries, the need for a VF or BF may be different. Therefore, the outcomes of these strategies for BPAI patients remained unknown thus far.

As the choice for initial management strategy in the studied institutions was mostly based on surgeon preference, we aimed to identify specific patient and injury characteristics that influenced that decision. Although we did not find significant differences between groups, specifically regarding associated tibiofemoral injuries such as the occurrence of tibial plateau fractures (*p* = 1.00), we speculate that specific injury characteristics have played a role in the decision to treat according to either the VF or BF strategy. This speculation, that lacks statistical power, is based on observed differences such as more high-energy mechanisms of injury in BF patients, a high median ISS in BF patients, and more severe tibial plateau fracture types in BF patients, which could imply more extensive orthopedic injuries to be present in BF patients.

Although median (IQR) total delay duration in the BF group of 6.8 h (4.0–7.5) was greater than the 5.5 h (4.0–8.5) in the VF group, total delay duration was not significantly different between groups (*p* = 1.00). Median (IQR) in-hospital delay in the BF group of 2.7 h (1.0–3.0) and the VF group of 2.6 h (2.0–2.9) was not different, as well (*p* = 0.82). When taking the total delay duration and population characteristics into account, we conclude that both the VF as well as the BF strategy have comparable good outcomes. We speculate that during treatment of BPAI patients with severe tibiofemoral fractures and dislocations, a BF strategy may be preferred in order to provide stability for the vascular repair. However, caution should be used when extrapolating these results to individual patients with other ischemia durations and total trauma burdens. In case of a longer total ischemic duration, irrespective of associated tibiofemoral injuries, a VF strategy (using intraluminal shunting if necessary) or a strategy where external fixation is preceded by vascular shunting before definite popliteal artery repair may be preferred, as total ischemia duration is a predictor for (secondary) amputation [10, 20].

### Long-term outcomes

Most patients received lower leg revascularization by popliteal artery bypass (59%) or interposition graft (33%), as transections and (severe) vessel-wall contusion limited endovascular treatment options. Therefore, these long-term outcomes are applicable to revascularization utilizing these surgical procedures. A total of 25 patients were available for long-term follow-up (93%), with a median (IQR) follow-up of 2.7 years (1.1–5.9). During this period, three vascular re-interventions were performed in these 25 patients, of which one was related to the reverse GSV bypass (4%). Excluding those that had re-interventions, no claudication complaints were observed. A total of 20 patients were started on ASA antiplatelet therapy (74%), mostly dosed 325 mg once daily, which was continued during the follow-up period. Using antiplatelet therapy, including ASA, as postoperative antithrombotic regimen to assure vascular patency, is in accordance with regimens of infrainguinal bypass surgery for (non-traumatic) peripheral arterial disease [25, 26]. Based on these results, we conclude that popliteal artery bypasses and interposition grafts for BPAI, using reversed GSV grafting in combination with antiplatelet therapy, appear to result in good vascular patency after a follow-up period of almost 3 years. Larger studies focusing on complications, re-interventions, and long-term vascular patency should be performed to determine if long-term outcomes are similar to the ones described.

### Limitations

Our study has several limitations. First, this was a retrospective study with a small number of patients. Although our results indicate comparably good outcomes between the VF and BF strategy for BPAI, larger studies are required to determine if (long-term) outcomes are similar to the ones we found. Of course, this is limited by the rare occurrence of these injuries and suggests that a multi-institutional effort may be needed for a larger study. In addition, the retrospective identification of patients during a 19-year period may potentially have resulted in missing cases.

Although no primary amputations were observed in our cohort, literature states that BPAIs are associated with high amputation rates. We speculate that this difference may be explained by undiagnosed BPAIs among the polytrauma patient population that did not survive emergency surgery (e.g., for intra-cranial or abdominal bleeding) and did not receive CTA scanning/peri-operative angiography (due to hemorrhagic shock). Polytrauma patients that required primary amputation of their mangled extremities due to the additional high total trauma burden may have had undiagnosed BPAIs, as well. This form of selection bias/survivorship bias may have resulted in better reported outcomes. Second, as patients received treatment according to the VF or BF strategy by surgeon preference (rather than randomization), selection bias is likely to be present. Although we speculate that patients with more severe orthopedic injuries were treated according to the BF strategy, we were unable to illustrate these differences with statistical significance. These differences may have been present and possibly have affected outcomes. Third, we were unable to accurately determine total ischemia duration, since the operative records did not literally mention the moment in time where an anastomosis was completed, and clamps were released. We, therefore, had to use total delay duration (time of injury—operating room arrival time) as the most accurate substitute for total ischemia duration. Fourth, as clinical outcomes of a VF or BF strategy for BPAI management are highly dependent on total ischemia duration (therefore ischemic damage to the lower leg), our results cannot be seen separate from the observed total delay durations. Caution should be used when extrapolating the results of both strategies to a setting with different (pre-)hospital trauma care logistics.

## Conclusion

BPAI is a rare and challenging surgical emergency. Both the VF and BF strategy are associated with good clinical outcomes for BPAI patients that received early revascularization after the initial injury. In case of extensive isolated orthopedic injury and knee instability, a BF strategy may be preferred if allowed by the total ischemia duration. Long-term outcomes of BPAI repairs appear to be good, independent of the initial management strategy.

## Supplementary Information

Below is the link to the electronic supplementary material.

Supplementary file1 (DOCX 31 KB)

## Data Availability

We have not filed for permission to publish the study material.
