# Supplementary material for: Blunt popliteal artery injury following tibiofemoral trauma: vessel-first and bone-first strategy
Source: Eur J Trauma Emerg Surg. 2021 Mar 20;48(2):1045–53. doi: 10.1007/s00068-021-01632-0 (PMC9001538; doi:10.1007/s00068-021-01632-0)
Supplement: Supplementary file 1 — Supplementary file1 (DOCX 31 KB) [file 68_2021_1632_MOESM1_ESM.docx]

**Table 1** Characteristics of all patients, those that were transported from an OSH and those transported from scene.

|  | **All patients** (n = 27) | **OSH** (n = 16) | **Scene** (n = 11) | **P-value** |
| --- | --- | --- | --- | --- |
| Age (years) | 38 [22 – 54] | 35 [19 – 47] | 51 [27 – 55] | 0.13 |
| Male | 15 (56) | 6 (38) | 9 (82) | 0.05* |
| BMI (kg/m^2^) | 36 [28 – 48] | 37 [27 – 54] | 34 [28 – 47] | 0.88 |
| Systolic blood pressure | 133 [118 – 155] | 132 [114 – 149] | 138 [118 – 163] | 0.49 |
| Hemodynamic instability | 4 (15) | 0 (0) | 4 (36) | 0.02* |
| Absent pulsations | 25 (93) | 15 (94) | 10 (91) | 1.00 |
| Inaudible doppler signal | 19 (70) | 10 (63) | 9 (82) | 0.40 |
| ISS | 9 [4 – 10] | 7 [4 – 9] | 9 [4 – 26] | 0.37 |
| Polytrauma | 5 (19) | 1 (6) | 4 (36) | 0.13 |
| Hb (mmol/L) | 8.3 [7.4 – 8.7] | 7.9 [6.8 – 8.3] | 9.1 [7.8 – 9.8] | < 0.01* |
| OSH vascular repair | 2 (7) | 2 (13) | 0 (0) | 0.50 |
| Total delay (hours)* | 5.7 [4.0 – 8.0] | 7.5 [5.7 – 10.0] | 4.0 [3.8 – 4.6] | < 0.01* |
| In-hospital diagnostic delay (hours) | 2.7 [2.0 – 3.0] | 2.7 [2.0 – 3.0] | 2.2 [2.0 – 2.7] | 0.35 |
| Severity of limb ischemia** |  |  |  | 1.00 |
| Viable (I) | 6 (22) | 4 (25) | 2 (18) |  |
| Threatened (II) | 18 (67) | 10 (63) | 8 (73) |  |
| Irreversible (III) | 3 (11) | 2 (13) | 1 (9) |  |

Data are presented as the number (%) or the median [IQR: 25th – 75th percentile]. BMI, Body Mass Index; ISS, Injury Severity Score; Hb, haemoglobin;
OSH, outside-hospital. *Total delay duration could not be accurately determined in 5 patients transported from an OSH and 3 patients transported from scene.
 **Using the (modified) Rutherford classification for acute limb ischemia. Percentages may not total 100 due to rounding.

**Table 2** Injury characteristics of all patients, those that were transported from an OSH and those transported from scene.

|  | **All patients** (n = 27) | **OSH** (n = 16) | **Scene** (n = 11) | **P-value** |
| --- | --- | --- | --- | --- |
| Mechanism of injury |  |  |  | 0.16 |
| Fall from height (<2m) | 8 (30) | 7 (44) | 1 (9) |  |
| Crush/Impact | 7 (26) | 3 (19) | 4 (36) |  |
| Fall from height (>2m) | 3 (11) | 2 (13) | 1 (9) |  |
| Pedestrian collision | 3 (11) | 1 (6) | 2 (18) |  |
| MVA | 2 (7) | 0 (0) | 2 (18) |  |
| Other | 4 (15) | 3 (19) | 1 (9) |  |
| Schenck knee dislocation grade* |  |  |  | 0.84 |
| I | 0 (0) | 0 (0) | 0 (0) |  |
| II | 3 (11) | 2 (13) | 1 (9) |  |
| III | 6 (22) | 3 (19) | 3 (27) |  |
| IV | 2 (7) | 2 (13) | 0 (0) |  |
| V | 7 (26) | 3 (19) | 4 (36) |  |
| N/a | 4 (15) | 3 (19) | 1 (9) |  |
| Popliteal artery injury |  |  |  | 0.85 |
| Dissection | 3 (11) | 2 (13) | 1 (9) |  |
| Occlusion | 18 (67) | 11 (69) | 7 (64) |  |
| Transection | 6 (22) | 3 (19) | 3 (27) |  |
| Previous knee injury | 5 (19) | 3 (19) | 2 (18) | 1.00 |
| Knee dislocation** | 23 (85) | 13 (81) | 10 (91) | 0.62 |
| Isolated ligamentous injuries | 14 (52) | 10 (63) | 4 (36) | 0.18 |
| Tibial plateau fracture | 9 (33) | 6 (38) | 3 (27) | 0.69 |
| Schatzker classification |  |  |  | 0.21 |
| Type 3 | 1 (1) | 0 (0) | 1 (9) |  |
| Type 4 | 4 (15) | 3 (50) | 1 (9) |  |
| Type 5 | 1 (4) | 0 (0) | 1 (9) |  |
| Type 6 | 3 (11) | 3 (50) | 0 (0) |  |
| Additional knee injuries |  |  |  |  |
| Distal femur fracture | 4 (15) | 1 (6) | 3 (27) | 0.27 |
| Fibula fracture | 6 (22) | 2 (13) | 4 (36) | 0.19 |
| Popliteal vein injury | 2 (7) | 1 (6) | 1 (9) | 1.00 |
| Tibial nerve injury | 1 (4) | 1 (6) | 0 (0) | 1.00 |

Data are presented as the number (%) or the median [IQR: 25th – 75th percentile]. MVA, motor vehicle accident. Percentages may not total 100 due to rounding.
*Due to the absence of MRI imaging records, Schenck knee dislocation grade could not be determined in 5 patients. **Including fracture dislocations.

**Table 3** Treatment characteristics and outcomes of all patients, those that were transported from an OSH and those transported from scene.

|  | **All patients** (n = 27) | **OSH** (n = 16) | **Scene** (n = 11) | **P-value** |
| --- | --- | --- | --- | --- |
| Popliteal artery reconstruction |  |  |  | 0.85 |
| Bypass | 16 (59) | 9 (56) | 7 (64) |  |
| Interposition graft | 9 (33) | 6 (38) | 3 (27) |  |
| Endovascular stenting | 2 (7) | 1 (6) | 1 (9) |  |
| Used reconstruction materials |  |  |  | 0.75 |
| Greater saphenous vein | 23 (85) | 13 (81) | 10 (91) |  |
| Synthetic graft | 2 (7) | 2 (13) | 0 (0) |  |
| Stent graft | 2 (7) | 1 (6) | 1 (9) |  |
| VF-strategy | 15 (56) | 10 (63) | 5 (45) | 0.38 |
| Surgical approach |  |  |  |  |
| Pre-operative angiography | 14 (52) | 7 (44) | 7 (64) | 0.31 |
| Medial approach | 23 (85) | 13 (81) | 10 (91) | 0.14 |
| Posterior approach | 3 (11) | 3 (19) | 0 (0) | 0.14 |
| Endovascular | 2 (7) | 1 (6) | 1 (9) |  |
| Proximal anastomosis site |  |  |  | 0.90 |
| PA | 16 (59) | 10 (63) | 6 (55) |  |
| SFA | 9 (33) | 5 (31) | 4 (36) |  |
| N/a | 2 (7) | 1 (6) | 1 (9) |  |
| Distal anastomosis site |  |  |  | 0.65 |
| PA | 19 (70) | 12 (75) | 7 (64) |  |
| PTA | 5 (19) | 3 (19) | 2 (18) |  |
| TPT | 1 (4) | 0 (0) | 1 (9) |  |
| N/a | 2 (7) | 1 (6) | 1 (9) |  |
| Additional procedures |  |  |  |  |
| Fasciotomy | 22 (81) | 14 (88) | 8 (73) | 0.37 |
| Shunting | 2 (7) | 1 (6) | 1 (9) | 1.00 |
| Additional orthopedic treatment |  |  |  |  |
| ORIF | 6 (22) | 2 (13) | 3 (27) | 0.66 |
| Ligament reconstruction | 8 (30) | 4 (25) | 4 (36) | 0.68 |
| Post-operative medical treatment |  |  |  | 0.38 |
| ASA | 20 (74) | 13 (81) | 7 (64) |  |
| Coumadin | 1 (4) | 0 (0) | 1 (9) |  |
| None | 6 (22) | 3 (19) | 3 (27) |  |
| Outcomes |  |  |  |  |
| Primary amputations | 0 (0) | 0 (0) | 0 (0) | 1.00 |
| Secondary amputations | 0 (0) | 0 (0) | 0 (0) | 1.00 |
| Pre-operative compartment syndrome | 3 (11) | 2 (13) | 1 (9) | 1.00 |
| Post-operative compartment syndrome | 0 (0) | 0 (0) | 0 (0) | 1.00 |
| Thrombo-embolic complications | 0 (0) | 0 (0) | 0 (0) | 1.00 |
| Post-operative infections | 3 (11) | 3 (19) | 0 (0) | 0.25 |
| Mortality | 0 (0) | 0 (0) | 0 (0) | 1.00 |
| Median (IQR) LOS (days) | 12 [7 – 21] | 13 [8 – 17] | 12 [7 – 31] | 0.44 |
| Median (IQR) ICU-LOS (days) | 0.5 [0 – 3] | 0 [0 – 2] | 1 [0 – 7] | 0.35 |
| Follow-up |  |  |  |  |
| Available follow-up | 25 (93) | 16 (100) | 9 (82) | 0.16 |
| Median (IQR) time to follow-up (years) | 2.7 [1.1 – 5.9] | 2.0 [0.6 – 3.7] | 3.8 [1.6 – 9.9] | 0.08 |
| Popliteal artery re-intervention | 3 (11) | 2 (13) | 1 (9) | 1.00 |
| Claudication | 0 (0) | 0 (0) | 0 (0) | 1.00 |

Data are presented as the number (%) or the median [IQR: 25th – 75th percentile]. VF, vessel-first; PA, popliteal artery; SFA, superficial femoral artery; PTA, posterior tibial artery;
TPT, tibioperoneal trunk; ORIF, open reduction internal fixation; ASA, acetylsalicylic acid; LOS; length of stay; ICU, intensive care unit.
Percentages may not total 100 due to rounding.
